# Therapeutic implications of cancer gene amplifications without mRNA overexpression: silence may not be golden

**DOI:** 10.1186/s13045-021-01211-1

**Published:** 2021-12-02

**Authors:** Amélie Boichard, Scott M. Lippman, Razelle Kurzrock

**Affiliations:** 1 Department of Molecular Cancer Genetics, University of Strasbourg Hospitals, 67000 Strasbourg, France; 2 Center for Personalized Cancer Therapy, Moores Cancer Center, University of California, La Jolla, CA 92093 USA; 3 WIN Consortium, Paris, France

**Keywords:** Gene amplification, mRNA expression, Therapeutic actionability

## Abstract

Amplifications of oncogenic genes are often considered actionable. However, not all patients respond. Questions have therefore arisen regarding the degree to which amplifications, especially non-focal ones, mediate overexpression. We found that a subset of high-level gene amplifications (≥ 6 copies) (from The Cancer Genome Atlas database) was not over-expressed at the RNA level. Unexpectedly, focal amplifications were more frequently silenced than non-focal amplifications. Most non-focal amplifications were not silenced; therefore, non-focal amplifications, if over-expressed, may be therapeutically tractable. Furthermore, specific silencing of high-level focal or non-focal gene amplifications may explain resistance to drugs that target the relevant gene product.

**Supplementary Information:**

The online version contains supplementary material available at 10.1186/s13045-021-01211-1.

## To the Editor,

Targeted therapy resistance affects a subset of cancer patients [1, 2]. Indeed, ~ 13% of somatic mutations are not expressed at the RNA level [3]. Little is known regarding gene amplifications. We examined The Cancer Genome Atlas (TCGA) to determine RNA expression of high-level amplifications, including in cancer-related genes. We observed that a minority of high-level amplifications are silenced. Silencing is, unexpectedly, more frequent in focal than non-focal amplifications. However, most focal and non-focal amplifications are over-expressed. Therefore, our observations suggest important points, which require further clinical validation: (1) high-level amplifications can be silenced and therefore may not be amenable to therapeutic targeting: and (2) non-focal amplifications are sometimes not considered druggable; however, they are frequently overexpressed, suggesting that they could be pharmacologically tractable.

Data from 675 TCGA samples (23 tumor types) with differential RNA expression was retrieved (Additional file 1: Methods ; Additional file 2: Table S1). A total of 166,707 amplifications were reviewed; there was an average of 304 [268–340] (mean [95% confidence interval (CI)]) high-level amplifications (≥ 6 copies) per sample. Amplifications were categorized as “non-focal” when ≥ 1 other amplification was observed within a 0.1 megabase (Mb) genomic window. Non-focal amplifications result from large genome rearrangements encompassing > 1 gene. Using the aforementioned threshold, 137,819 (83%) amplifications were considered non-focal; 28,888 (17%) amplifications, focal; respectively, 5612 non-focal (68%) and 2599 focal (32%) amplifications in cancer-related genes (Table 1).

**Table 1:** Silencing distribution between focal and non-focal high-level amplifications (data from TCGA)

	Total number of high-level amplifications*	Tumor-to-normal differential RNA expression		Number of silenced** amplifications	Number of non-silenced amplifications	Chi-square *p* value
	*N* (%)	Mean [95% confidence interval]	*T* test *p* value	*N* (%)	*N* (%)	
All genes (*N* = 18,870)						
All amplifications	166,707 (100%)	+ 548 [+ 500 to + 595] %	–	9534 (6%)	157,173 (94%)	–
Focal amplifications	137,819 (83%)	+ 559 [+ 505 to + 613] %	0.296	6973 (4%)	130,846 (78%)	**< 0.00001**
Non-focal amplifications***	28,888 (17%)	+ 493 [+ 400 to + 585] %		**2561 (2%)**	26,328 (16%)	
Cancer-related genes**** (*N* = 832)						
All amplifications	8211 (100%)	+ 1084 [+ 742 to + 1426] %	–	485 (6%)	7726 (94%)	–
Focal amplifications	5612 (68%)	+ 1207 [+ 723 to + 1691] %	0.300	233 (3%)	5379 (66%)	**< 0.00001**
Non-focal amplifications	2599 (32%)	+ 818 [+ 549 to + 1088] %		**252 (3%)**	2347 (29%)	

Numbers in bold represent statistically significant Chi-square p-values at the alpha level of 0.05, as well as the criteria that have the largest contribution to the Chi-square statistic

*Gene level amplification included those genes with ≥ 6 copies

**RNA silencing was defined by an 80% decrease of expression in the tumor sample compared to the normal sample, including only tumor samples that presented a high-level amplification for that gene (≥ 6 copies of the gene)

***Non-focal amplifications are co-amplification of genes that are located in the same 0.1 megabase genomic window

****Cancer-related genes are listed in Additional file 2: Table S2. The list of cancer-related genes was defined as the union of genes curated by the Cancer Gene Census (CGC) from the Catalogue of Somatic Mutations in Cancer (COSMIC) and genes analyzed by Foundation Medicine Inc. in their commercial panels Foundation One and Foundation One Heme (*N* = 946 distinct genes)

High-level amplifications correlated with a + 548 [+ 500 to + 595] % (mean, 95% CI) increase of expression for the corresponding mRNA in the tumor compared to the adjacent tissue. When considering only cancer-related genes, the overall expression increase was + 1084 [+ 742 to + 1426]% (mean, 95% CI). There was no difference between the focal and the non-focal groups (Table 1).

A subset of 9534 (6%) amplifications were silenced (i.e., presenting a decrease of expression > 80% in the tumor compared to normal adjacent tissue). This proportion was similar when only considering cancer-related genes (*N* = 485, 6%) (Table 1).

**Fig. 1:**
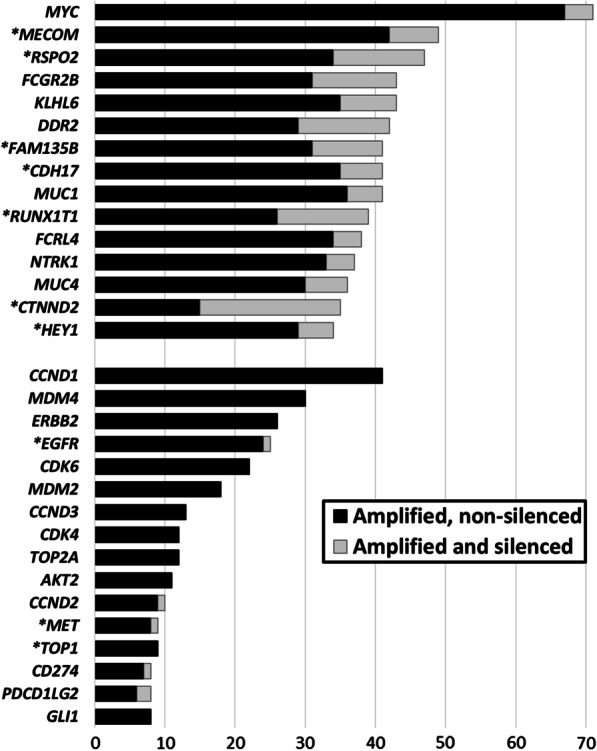
Frequently silenced cancer-related gene amplifications (TCGA). Top panel: cancer-related genes that are amplified in ≥ 5% (*N* ≥ 34) of the tumor samples (*N* = 675) and silenced in ≥ 10% of those cases (*N* ≥ 4). Black = amplified, not silenced; grey = amplified and silenced. Bottom panel (starting with *CCND1*): sampling of common potentially actionable oncogenes; they are infrequently silenced. *Genes preceded by an asterisk were exclusively found to be focally amplified. Gene level amplification included those genes with ≥ 6 copies; RNA silencing was defined by ≥ 80% expression decrease in the tumor sample compared to the normal sample, including only tumor samples that had high-level amplification for that gene (≥ 6 copies of the gene); non-focal amplifications referred to co-amplification of genes located in the same 0.1 megabase genomic window

A Chi-square test was performed to examine the relationship between gene silencing and amplification type. Interestingly, gene amplifications were either consistently focal or non-focal. Amongst the 832 amplified cancer-related genes, 243 were always focally amplified and 589 were uniformly non-focally amplified. Focal amplifications were more likely to be silenced (9% vs 5% silenced amplifications; odds-ratio (OR) [95% CI] = 1.83 [1.74–1.91]; *X*^2^ (1, *N* = 166,707) = 641.4, *p* < 0.00001), and this held true when considering only cancer-related genes (10% versus 4% silenced amplifications; OR = 2.48 [2.06–2.98]; *X*^2^ (1, *N* = 8211) = 98.2, *p* < 0.00001) (Table 1). Interestingly, many cancer-related genes that are druggable/activate druggable pathways were less frequently silenced than cancer-related genes without active therapies (Fig. 1).

The mechanism of amplification silencing was not elucidated. Prior studies suggest potential mechanisms such as epigenetic modifications [4], miRNA regulation [5], and/or factors that influence RNA-decay such as RNA-binding proteins [6].

Overall, our findings indicate that high-level amplifications are prevalent in tumors. Further, as expected, gene copy-number amplifications correlated with an overall increase in mRNA expression. Even so, high-level amplifications may be associated with gene-specific silenced RNA expression. Surprisingly, focal amplifications were more likely to be silenced than non-focal amplifications, and this held true when considering only cancer-related genes. Furthermore, most non-focal amplifications were not silenced, suggesting that such amplifications may still be actionable. Of interest, cancer genes that were more likely to be considered druggable and/or have established prognostic or predictive attributes were usually not silenced, while cancer-related genes that are often considered therapeutically intractable were more often silenced. Since silencing of amplifications would nullify tumorigenic impact, and also lead to resistance (if the gene product was the treatment target), it is conceivable that more frequently silenced amplifications would be less likely to be associated with therapeutic or prognostic/predictive impact. These findings echo those previously published in gliomas where the authors suggested that, even when amplified, genes that are normally silent in a given cell type may play no role in tumor progression [7].

In conclusion, our study indicates that the consequences of silencing on response versus resistance after targeted therapies matched to oncogenic amplifications requires in vitro verification and prospective clinical studies. Taken together with the existing literature [3, 8, 9], we suggest that gene silencing may be an important mechanism of therapeutic resistance, and that optimal pharmacologic intervention in cancer may demand transcriptomic in addition to genomic interrogation and considerations for epigenetic modulation.

## Supplementary Information

**Additional file 1.** Methods [10–13].

**Additional file 2.** Supplementary Tables 1 and 2.

## Data Availability

The data that support the findings of this study derived from The Cancer Genome Atlas (TCGA) project (https://www.cancer.gov/about-nci/organization/ccg/research/structural-genomics/tcga).

## Abbreviations

CGC: Cancer Gene Census
CI: Confidence interval
CNV: Copy number variation
COSMIC: Catalogue Of Somatic Mutation In Cancer
GISTIC: Genomic Identification of Significant Targets In Cancer
Mb: Megabase
N: Number
OR: Odds ratio
RNA/mRNA: Ribonucleic acid/messenger ribonucleic acid
RSEM: RNA-seq by expectation–maximisation
TCGA: The Cancer Genome Atlas
UCSC: University of California Santa Cruz
