# Supplementary material for: Therapeutic implications of cancer gene amplifications without mRNA overexpression: silence may not be golden
Source: J Hematol Oncol. 2021 Dec 2;14:201. doi: 10.1186/s13045-021-01211-1 (PMC8638100; doi:10.1186/s13045-021-01211-1)
Supplement: Supplementary file 1 — Additional file 1. Methods [10–13]. [file 13045_2021_1211_MOESM1_ESM.docx]

**METHODS (Supplementary data)**

***The Cancer Genome Atlas (TCGA) data sets***

Somatic gene-level copy number variations (CNVs) and RNA-sequencing expression data from all TCGA samples (N = 10,845 and 10,535 samples respectively, 34 different cohorts) where downloaded from UCSC Xena data portal (<https://xena.ucsc.edu/>).

Genomic copy numbers were measured experimentally using whole-genome microarray. Gene-level CNVs were normalized using data from all TCGA cohorts (« pan-cancer » data set) and estimated using the GISTIC2 threshold method(10), where the values -2, -1, 0, 1 and 2 are representing homozygous deletion, single-copy deletion, diploid normal copy, low-level amplification, and high-level amplification, respectively. Genes were mapped to the human genome coordinates using UCSC cgData HUGO probeMap (hg18). Copy-number estimates were available for 24,777 genes on 10,845 samples (UCSC Xena analysis version 2016-08-16). Only high-level amplifications were considered for the analysis. High-level amplifications were considered non-focal when the genomic distance between two amplified genes was shorter than 0.1 Mb.

Whole RNA expression was measured experimentally using RNA-sequencing, estimated using the RSEM method(11) and normalized between TCGA cohorts using the UCSC Toil RNA-seq Recompute tool(12). Transcripts/genes were mapped to the human genome coordinates using UCSC cgData HUGO probeMap (hg18). mRNA expression normalized estimates (expressed as log2(norm_count+1)) were available for 58,582 genes (including protein-coding and noncoding genes) on 10,535 samples (UCSC Xena analysis version 2016-02-18). Differential RNA expression was calculated by comparing RNA expression level obtained in normal adjacent tissue compared to the level obtained in tumor sample (primary tumor or metastasis). Only samples for which tumor-to-normal differential expression was available were kept for the analysis (N = 675). mRNA silencing was defined by an 80% decrease of expression in the tumor sample compared to the normal sample, including only tumor samples that had at least high-level amplification for that gene. Copy number amplification was > 6(13). The list of cancer-related genes was defined as the union of genes curated by the Cancer Gene Census (CGC) from the Catalogue of Somatic Mutations in Cancer (COSMIC) and genes analyzed by Foundation Medicine in their commercial panels Foundation One and Foundation One Heme (N = 946 distinct genes).

***Statistical analysis***

A T-test was used to assess differences of mRNA expression levels between groups; while a Chi-square test was used to assess differences of distribution of high-level amplifications between groups.
