# Supplementary material for: Therapeutic implications of cancer gene amplifications without mRNA overexpression: silence may not be golden
Source: J Hematol Oncol. 2021 Dec 2;14:201. doi: 10.1186/s13045-021-01211-1 (PMC8638100; doi:10.1186/s13045-021-01211-1)
Supplement: Supplementary file 2 — Additional file 2. Supplementary Tables 1 and 2. [file 13045_2021_1211_MOESM2_ESM.docx]

**Supplementary Table 1: Prevalence of high-level (>6 copies) amplifications in human tumors from The Cancer Genome Atlas (N= 675 tumor specimens with >1 gene amplification and available normal versus tumor expression data) (data from TCGA).**

| **TCGA cohort** | **Number of samples**  **analyzed*** | **Number of high-level (>6 copies) amplifications per sample**  **(average)** |
| --- | --- | --- |
| Sarcoma | 2 | 544 |
| Breast invasive carcinoma | 110 | 539 |
| Hepatocellular carcinoma | 50 | 512 |
| Esophageal carcinoma | 13 | 472 |
| Lung squamous cell carcinoma | 50 | 468 |
| Bladder Urothelial Carcinoma | 19 | 426 |
| Rectum adenocarcinoma | 6 | 418 |
| Lung adenocarcinoma | 56 | 410 |
| Cholangiocarcinoma | 9 | 382 |
| Pancreatic adenocarcinoma | 4 | 365 |
| Head & neck squamous cell carcinoma | 43 | 364 |
| Stomach adenocarcinoma | 33 | 348 |
| Uterine corpus endometrial carcinoma | 7 | 318 |
| Melanoma | 1 | 307 |
| Thymoma | 2 | 172 |
| Renal clear cell carcinoma | 71 | 163 |
| Cervical squamous cell carcinoma and endocervical adenocarcinoma | 3 | 159 |
| Colon adenocarcinoma | 26 | 141 |
| Prostate adenocarcinoma | 52 | 55 |
| Kidney renal papillary cell carcinoma | 32 | 54 |
| Kidney chromophobe | 25 | 40 |
| Pheochromocytoma and paraganglioma | 3 | 11 |
| Thyroid carcinoma | 58 | 1 |

** Includes only those patients in TCGA for whom tumor-to-normal differential RNA expression was available.*

**Supplementary Table 2: Cancer related genes considered (see Table 1).**

The list of cancer-related genes was defined as the union of genes curated by the Cancer Gene Census (CGC) from the Catalogue of Somatic Mutations in Cancer (COSMIC) and genes analyzed by Foundation Medicine Inc. in their commercial panels Foundation One and Foundation One Heme (N = 946 distinct genes).

| *A1CF* | *CAMTA1* | *DAXX* | *FGF12* | *HRAS* | *MAP3K1* | *NSD2* | *PTPN2* | *SND1* | U2AF1 |
| --- | --- | --- | --- | --- | --- | --- | --- | --- | --- |
| *ABI1* | *CANT1* | *DCAF12L2* | *FGF14* | *HSD3B1* | *MAP3K13* | *NSD3* | *PTPN6* | *SNX29* | U2AF2 |
| *ABL1* | *CARD11* | *DCC* | *FGF19* | *HSP90AA1* | *MAP3K14* | *NT5C2* | *PTPRB* | *SOCS1* | UBR5 |
| *ABL2* | *CARS* | *DCTN1* | *FGF23* | *HSP90AB1* | *MAP3K6* | *NTHL1* | *PTPRC* | *SOCS2* | USP44 |
| *ACKR3* | *CASP3* | *DDB2* | *FGF3* | *ICK* | *MAP3K7* | *NTRK1* | *PTPRD* | *SOCS3* | USP6 |
| *ACSL3* | *CASP8* | *DDIT3* | *FGF4* | *ID3* | *MAPK1* | *NTRK2* | *PTPRK* | *SOX10* | USP8 |
| *ACSL6* | *CASP9* | *DDR1* | *FGF6* | *IDH1* | *MAX* | *NTRK3* | *PTPRO* | *SOX2* | VAV1 |
| *ACTB* | *CBFA2T3* | *DDR2* | *FGFR1* | *IDH2* | *MB21D2* | *NUMA1* | *PTPRT* | *SOX21* | VEGFA |
| *ACVR1* | *CBFB* | *DDX10* | *FGFR1OP* | *IGF1R* | *MCL1* | *NUP214* | *PWWP2A* | *SOX9* | VHL |
| *ACVR1B* | *CBL* | *DDX3X* | *FGFR2* | *IGF2BP2* | *MDM2* | *NUP93* | *QKI* | *SPECC1* | VTI1A |
| *ACVR2A* | *CBLB* | *DDX5* | *FGFR3* | *IGH* | *MDM4* | *NUP98* | *RABEP1* | *SPEN* | WAS |
| *AFDN* | *CBLC* | *DDX6* | *FGFR4* | *IGK* | *MDS2* | *NUTM1* | *RAC1* | *SPOP* | WDCP |
| *AFF1* | *CCDC6* | *DEK* | *FH* | *IGL* | *MECOM* | *NUTM2B* | *RAD17* | *SRC* | WDR90 |
| *AFF3* | *CCNB1IP1* | *DGCR8* | *FHIT* | *IGLJAK1* | *MED12* | *NUTM2D* | *RAD21* | *SRGAP3* | WHSC1 |
| *AFF4* | *CCNC* | *DICER1* | *FIP1L1* | *IKBKB* | *MEF2B* | *OLIG2* | *RAD50* | *SRSF2* | WHSC1L1 |
| *AKAP9* | *CCND1* | *DIS3* | *FKBP9* | *IKBKE* | *MEF2C* | *OMD* | *RAD51* | *SRSF3* | WIF1 |
| *AKT1* | *CCND2* | *DNAJB1* | *FLCN* | *IKZF1* | *MEK1* | *P2RY8* | *RAD51B* | *SS18* | WISP3 |
| *AKT2* | *CCND3* | *DNM2* | *FLI1* | *IKZF2* | *MEK2* | *PABPC1* | *RAD51C* | *SS18L1* | WNK2 |
| *AKT3* | *CCNE1* | *DNMT3A* | *FLNA* | *IKZF3* | *MEN1* | *PAFAH1B2* | *RAD51D* | *SSX1* | WRN |
| *ALDH2* | *CCR4* | *DOT1L* | *FLT1* | *IL2* | *MERTK* | *PAG1* | *RAD52* | *SSX2* | WT1 |
| *ALK* | *CCR7* | *DROSHA* | *FLT3* | *IL21R* | *MET* | *PAK3* | *RAD54L* | *SSX4* | WTX |
| *ALOX12B* | *CCT6B* | *DTX1* | *FLT4* | *IL6ST* | *MGMT* | *PALB2* | *RAF1* | *STAG1* | WWTR1 |
| *AMER1* | *CD209* | *DUSP2* | *FLYWCH1* | *IL7R* | *MIB1* | *PARK2* | *RALGDS* | *STAG2* | XBP1 |
| *ANK1* | *CD22* | *DUSP9* | *FNBP1* | *INHBA* | *MITF* | *PARP1* | *RANBP2* | *STAT3* | XPA |
| *APC* | *CD274* | *DUX4L1* | *FOXA1* | *INPP4B* | *MKI67* | *PARP2* | *RAP1GDS1* | *STAT4* | XPC |
| *APH1A* | *CD28* | *E2A* | *FOXL2* | *INPP5D* | *MKNK1* | *PARP3* | *RARA* | *STAT5A* | XPO1 |
| *APOBEC3B* | *CD36* | *EBF1* | *FOXO1* | *IRF1* | *MLF1* | *PASK* | *RASGEF1A* | *STAT5B* | XRCC2 |
| *AR* | *CD58* | *ECT2L* | *FOXO3* | *IRF2* | *MLH1* | *PATZ1* | *RB1* | *STAT6* | YWHAE |
| *ARAF* | *CD70* | *EED* | *FOXO4* | *IRF4* | *MLL* | *PAX3* | *RBM10* | *STIL* | YY1AP1 |
| *ARFRP1* | *CD74* | *EGFR* | *FOXP1* | *IRF8* | *MLL2* | *PAX5* | *RBM15* | *STK11* | ZBTB16 |
| *ARHGAP26* | *CD79A* | *EIF1AX* | *FOXR1* | *IRS2* | *MLL3* | *PAX7* | *RECQL4* | *STRN* | ZCCHC8 |
| *ARHGAP5* | *CD79B* | *EIF3E* | *FRS2* | *IRS4* | *MLLT1* | *PAX8* | *REL* | *SUFU* | ZEB1 |
| *ARHGEF10* | *CDC73* | *EIF4A2* | *FSTL3* | *ISX* | *MLLT10* | *PBRM1* | *RELN* | *SUZ12* | ZFHX3 |
| *ARHGEF10L* | *CDH1* | *ELF3* | *FUBP1* | *ITGAV* | *MLLT11* | *PBX1* | *RET* | *SYK* | ZMYM2 |
| *ARHGEF12* | *CDH10* | *ELF4* | *FUS* | *ITK* | *MLLT3* | *PC* | *RFWD3* | *TAF1* | ZMYM3 |
| *ARID1A* | *CDH11* | *ELK4* | *GABRA6* | *JAK1* | *MLLT6* | *PCBP1* | *RGPD3* | *TAF15* | ZNF217 |
| *ARID1B* | *CDH17* | *ELL* | *GADD45B* | *JAK2* | *MMSET* | *PCLO* | *RGS7* | *TAL1* | ZNF24 |
| *ARID2* | *CDK12* | *ELN* | *GAS7* | *JAK3* | *MN1* | *PCM1* | *RHOA* | *TAL2* | ZNF331 |
| *ARNT* | *CDK4* | *ELP2* | *GATA1* | *JARID2* | *MNX1* | *PD-1* | *RHOH* | *TBL1XR1* | ZNF384 |
| *ASMTL* | *CDK6* | *EML4* | *GATA2* | *JAZF1* | *MPL* | *PDCD1* | *RICTOR* | *TBX3* | ZNF429 |
| *ASPSCR1* | *CDK8* | *EMSY* | *GATA3* | *JUN* | *MRE11A* | *PDCD11* | *RMI2* | *TCEA1* | ZNF479 |
| *ASXL1* | *CDKN1A* | *EP300* | *GATA4* | *KAT6A* | *MRTFA* | *PDCD1LG2* | *RNF213* | *TCF12* | ZNF521 |
| *ASXL2* | *CDKN1B* | *EPAS1* | *GATA6* | *KAT6B* | *MSH2* | *PDE4DIP* | *RNF43* | *TCF3* | ZNF703 |
| *ATF1* | *CDKN2A* | *EPHA3* | *GID4* | *KAT7* | *MSH3* | *PDGFB* | *ROBO2* | *TCF7L2* | ZNRF3 |
| *ATIC* | *CDKN2B* | *EPHA5* | *GLI1* | *KCNJ5* | *MSH6* | *PDGFRA* | *ROS1* | *TCL1* | ZRSR2 |
| *ATM* | *CDKN2C* | *EPHA7* | *GMPS* | *KDM2B* | *MSI2* | *PDGFRB* | *RPL10* | *TCL1A* | ZSCAN3 |
| *ATP1A1* | *CDX2* | *EPHB1* | *GNA11* | *KDM4C* | *MSN* | *PDK1* | *RPL22* | *TEC* |  |
| *ATP2B3* | *CEBPA* | *EPHB4* | *GNA12* | *KDM5A* | *MST1R* | *PD-L1* | *RPL5* | *TEK* |  |
| *ATR* | *CEP89* | *EPOR* | *GNA13* | *KDM5C* | *MTAP* | *PD-L2* | *RPN1* | *TENT5C* |  |
| *ATRX* | *CHCHD7* | *EPS15* | *GNAQ* | *KDM6A* | *MTCP1* | *PER1* | *RPTOR* | *TERC* |  |
| *AURKA* | *CHD2* | *ERBB2* | *GNAS* | *KDR* | *MTOR* | *PHF6* | *RSPO2* | *TERT* |  |
| *AURKB* | *CHD4* | *ERBB3* | *GOLGA5* | *KDSR* | *MUC1* | *PHOX2B* | *RSPO3* | *TET1* |  |
| *AXIN1* | *CHEK1* | *ERBB4* | *GOPC* | *KEAP1* | *MUC16* | *PICALM* | *RUNX1* | *TET2* |  |
| *AXIN2* | *CHEK2* | *ERC1* | *GPC3* | *KEL* | *MUC4* | *PIK3C2B* | *RUNX1T1* | *TFE3* |  |
| *AXL* | *CHIC2* | *ERCC2* | *GPC5* | *KIAA1549* | *MUTYH* | *PIK3C2G* | *S100A7* | *TFEB* |  |
| *B2M* | *CHST11* | *ERCC3* | *GPHN* | *KIF5B* | *MYB* | *PIK3CA* | *S1PR2* | *TFG* |  |
| *BACH1* | *CIC* | *ERCC4* | *GPR124* | *KIT* | *MYC* | *PIK3CB* | *SALL4* | *TFPT* |  |
| *BAP1* | *CIITA* | *ERCC5* | *GRAF* | *KLF4* | *MYCL* | *PIK3CG* | *SBDS* | *TFRC* |  |
| *BARD1* | *CKS1B* | *ERG* | *GRIN2A* | *KLF6* | *MYCL1* | *PIK3R1* | *SDC4* | *TGFBR2* |  |
| *BAX* | *CLIP1* | *ERRFI1* | *GRM3* | *KLHL6* | *MYCN* | *PIK3R2* | *SDHA* | *THRAP3* |  |
| *BAZ1A* | *CLP1* | *ESR1* | *GSK3B* | *KLK2* | *MYD88* | *PIM1* | *SDHAF2* | *TIPARP* |  |
| *BCL10* | *CLTC* | *ETNK1* | *GTSE1* | *KMT2A* | *MYH11* | *PLAG1* | *SDHB* | *TLL2* |  |
| *BCL11A* | *CLTCL1* | *ETS1* | *H3F3A* | *KMT2C* | *MYH9* | *PLCG1* | *SDHC* | *TLX1* |  |
| *BCL11B* | *CNBD1* | *ETV1* | *H3F3B* | *KMT2D* | *MYO18A* | *PLCG2* | *SDHD* | *TLX3* |  |
| *BCL2* | *CNBP* | *ETV4* | *HDAC1* | *KNL1* | *MYO5A* | *PML* | *SEPT5* | *TMEM127* |  |
| *BCL2L1* | *CNOT3* | *ETV5* | *HDAC4* | *KNSTRN* | *MYOD1* | *PMS1* | *SEPT6* | *TMEM30A* |  |
| *BCL2L12* | *CNTNAP2* | *ETV6* | *HDAC7* | *KRAS* | *MYST3* | *PMS2* | *SEPT9* | *TMPRSS2* |  |
| *BCL2L2* | *CNTRL* | *EWSR1* | *HERPUD1* | *KTN1* | *N4BP2* | *POLD1* | *SERP2* | *TMSB4XP8* |  |
| *BCL3* | *COL1A1* | *EXOSC6* | *HEY1* | *LARP4B* | *NAB2* | *POLE* | *SET* | *TMSL3* |  |
| *BCL6* | *COL2A1* | *EXT1* | *HGF* | *LASP1* | *NACA* | *POLG* | *SETBP1* | *TNC* |  |
| *BCL7A* | *COL3A1* | *EXT2* | *HIF1A* | *LATS1* | *NBEA* | *POLQ* | *SETD1B* | *TNFAIP3* |  |
| *BCL9* | *COX6C* | *EZH2* | *HIP1* | *LATS2* | *NBN* | *POT1* | *SETD2* | *TNFRSF11A* |  |
| *BCL9L* | *CPEB3* | *EZR* | *HIST1H1C* | *LCK* | *NCKIPSD* | *POU2AF1* | *SETDB1* | *TNFRSF14* |  |
| *BCLAF1* | *CPS1* | *FAF1* | *HIST1H1D* | *LCP1* | *NCOA1* | *POU5F1* | *SF3B1* | *TNFRSF17* |  |
| *BCOR* | *CREB1* | *FAM123B* | *HIST1H1E* | *LEF1* | *NCOA2* | *PPARG* | *SFPQ* | *TNFRSF6* |  |
| *BCORL1* | *CREB3L1* | *FAM131B* | *HIST1H2AC* | *LEPROTL1* | *NCOA4* | *PPFIBP1* | *SFRP4* | *TOP1* |  |
| *BCR* | *CREB3L2* | *FAM135B* | *HIST1H2AG* | *LHFPL6* | *NCOR1* | *PPM1D* | *SGK1* | *TP53* |  |
| *BIRC3* | *CREBBP* | *FAM46C* | *HIST1H2AL* | *LIFR* | *NCOR2* | *PPP2R1A* | *SH2B3* | *TP63* |  |
| *BIRC6* | *CRKL* | *FAM47C* | *HIST1H2AM* | *LMNA* | *NCSTN* | *PPP2R2A* | *SH3GL1* | *TPM3* |  |
| *BLM* | *CRLF2* | *FANCA* | *HIST1H2BC* | *LMO1* | *NDRG1* | *PPP6C* | *SHIP* | *TPM4* |  |
| *BMP5* | *CRNKL1* | *FANCC* | *HIST1H2BJ* | *LMO2* | *NF1* | *PRCC* | *SHP-1* | *TPR* |  |
| *BMPR1A* | *CRTC1* | *FANCD2* | *HIST1H2BK* | *LPP* | *NF2* | *PRDM1* | *SHTN1* | *TRA* |  |
| *BRAF* | *CRTC3* | *FANCE* | *HIST1H2BO* | *LRIG3* | *NFATC2* | *PRDM16* | *SIRPA* | *TRAF2* |  |
| *BRCA1* | *CSF1R* | *FANCF* | *HIST1H3B* | *LRP1B* | *NFE2L2* | *PRDM2* | *SIX1* | *TRAF3* |  |
| *BRCA2* | *CSF3R* | *FANCG* | *HIST1H4I* | *LRRK2* | *NFIB* | *PREX2* | *SIX2* | *TRAF5* |  |
| *BRD3* | *CSMD3* | *FANCL* | *HLA-A* | *LSM14A* | *NFKB2* | *PRF1* | *SKI* | *TRAF7* |  |
| *BRD4* | *CTCF* | *FAS* | *HLF* | *LTK* | *NFKBIA* | *PRKACA* | *SLC34A2* | *TRB* |  |
| *BRIP1* | *CTNNA1* | *FAT1* | *HMGA1* | *LYL1* | *NFKBIE* | *PRKAR1A* | *SLC45A3* | *TRD* |  |
| *BRSK1* | *CTNNA2* | *FAT3* | *HMGA2* | *LYN* | *NIN* | *PRKCB* | *SMAD2* | *TRG* |  |
| *BTG1* | *CTNNB1* | *FAT4* | *HMGN2P46* | *LZTR1* | *NKX2-1* | *PRKCI* | *SMAD3* | *TRIM24* |  |
| *BTG2* | *CTNND1* | *FBLN2* | *HNF1A* | *MACC1* | *NOD1* | *PRKDC* | *SMAD4* | *TRIM27* |  |
| *BTK* | *CTNND2* | *FBXO11* | *HNRNPA2B1* | *MAF* | *NONO* | *PRPF40B* | *SMARCA1* | *TRIM33* |  |
| *BTLA* | *CUL3* | *FBXO31* | *HOOK3* | *MAFB* | *NOTCH1* | *PRRX1* | *SMARCA4* | *TRIP11* |  |
| *BUB1B* | *CUL4A* | *FBXW7* | *HOXA11* | *MAGED1* | *NOTCH2* | *PRSS8* | *SMARCB1* | *TRRAP* |  |
| *C11orf30* | *CUX1* | *FCGR2B* | *HOXA13* | *MALAT1* | *NOTCH3* | *PSIP1* | *SMARCD1* | *TSC1* |  |
| *C15orf65* | *CXCR4* | *FCRL4* | *HOXA9* | *MALT1* | *NPM1* | *PTCH1* | *SMARCE1* | *TSC2* |  |
| *C17orf39* | *CYLD* | *FEN1* | *HOXC11* | *MAML2* | *NR4A3* | *PTEN* | *SMC1A* | *TSHR* |  |
| *CACNA1D* | *CYP17A1* | *FES* | *HOXC13* | *MAP2K1* | *NRAS* | *PTK6* | *SMC3* | *TUSC3* |  |
| *CAD* | *CYP2C8* | *FEV* | *HOXD11* | *MAP2K2* | *NRG1* | *PTPN11* | *SMO* | *TYK2* |  |
| *CALR* | *CYSLTR2* | *FGF10* | *HOXD13* | *MAP2K4* | *NSD1* | *PTPN13* | *SNCAIP* | *TYRO3* |  |
